# NACC1, as a Target of MicroRNA-331-3p, Regulates Cell Proliferation in Urothelial Carcinoma Cells

**DOI:** 10.3390/cancers10100347

**Published:** 2018-09-21

**Authors:** Kohei Morita, Tomomi Fujii, Hiroe Itami, Tomoko Uchiyama, Tokiko Nakai, Kinta Hatakeyama, Aya Sugimoto, Makito Miyake, Yasushi Nakai, Nobumichi Tanaka, Keiji Shimada, Masaharu Yamazaki, Kiyohide Fujimoto, Chiho Ohbayashi

**Affiliations:** 1 Department of Diagnostic Pathology, Nara Medical University School of Medicine, Nara 634-8521, Japan; moritak@naramed-u.ac.jp (K.M.); hritami@naramed-u.ac.jp (H.I.); uchiyama0403@naramed-u.ac.jp (T.U.); tokiko@naramed-u.ac.jp (T.N.); kpathol@naramed-u.ac.jp (K.H.); ayaasano1018@yahoo.co.jp (A.S.); ohbayashi@naramed-u.ac.jp (C.O.); 2 Department of Urology, Nara Medical University School of Medicine, Nara 634-8521, Japan; makitomiyake@naramed-u.ac.jp (M.M.); nakaiyasusiuro@live.jp (Y.N.); sendo@naramed-u.ac.jp (N.T.); kiyokun@naramed-u.ac.jp (K.F.); 3 Department of Diagnostic Pathology, Nara City Hospital, Nara 634-8521, Japan; k-shimada@nara-jadecom.jp; 4 Department of Central Clinical Laboratory, Nara Medical University Hospital, Nara 634-8521, Japan; masayama@naramed-u.ac.jp

**Keywords:** urothelial carcinoma, NACC1, miR-331-3p, cell cycle arrest, migration and invasion

## Abstract

The nucleus accumbens-associated protein 1 (NACC1) is a transcription factor constitutively expressed in the urothelium, where it regulates cell growth, senescence, autophagy, and epithelial-mesenchymal transition. microRNA (miRNA) constitutes a class of small non-coding RNAs which are involved in cell proliferation, differentiation, and progression of tumors. miRNAs and their target molecules are utilized for molecular diagnosis of urothelial carcinoma. NACC1 is one of several putative target molecules of miR-331-3p, and is associated with cell proliferation in cancers such as prostate and cervical cancer. Functional experiments involving miR-331-3p and its target molecule NACC1 were conducted using the urothelial carcinoma (UC) cell lines, T24, UMUC6, and KU7. Furthermore, quantitative reverse transcription polymerase chain reaction and immunostaining were performed to evaluate the expression of NACC1 in UC derived from transurethral resection of bladder tumor (TUR-Bt) specimens. The methane thiosulfonate (MTS) assay revealed that cell proliferation was significantly reduced after transient transfection of miR-331-3p precursor and/or NACC1 siRNA in UC cells. Cell senescence via cell cycle arrest at the G1 phase was induced by NACC1 inhibition. On the other hand, suppression of NACC1 induced cell migration and invasion abilities. Immunohistochemical analysis of TUR-Bt specimens revealed that over 70% of UC cells presented strongly positive results for NACC1. In contrast, normal urothelial cells were weakly positive for NACC1. It was also found that NACC1 expression was lower in invasive UC cells than in non-invasive UC cells. Loss of NACC1 induced vessel invasion in invasive UC tissues. The present results indicate that NACC1 regulated by miR-331-3p contributes to cell proliferation, and is involved in cell migration and invasion. This suggests that NACC1 can serve as a potential target molecule for the prediction and prognosis of UC, and can contribute to effective treatment strategies.

## 1. Introduction

Urothelial carcinoma (UC) typically occurs in the urinary system and is the most common type of bladder cancer. UC was estimated to be the fourth most prevalent of all new cancer diagnoses among US males in 2016 [1]. The incidence rate of bladder cancer is approximately four times higher in men than in women; the mortality rate is estimated to be 2–10/100,000 deaths per year in men and 0.5–4/100,000 in women [1]. Despite advances in pathological diagnosis and clinical treatment, including transurethral resection of bladder tumors (TUR-Bt) or intravesical instillation of bacillus calmette-guérin (BCG), UC remains a major cause of cancer-related morbidity and mortality. UC constitutes approximately 90% of urinary bladder cancer and is classified into two types, high-grade invasive and low-grade non-invasive carcinomas. Tumor invasion of the muscularis propria is one of the most important prognostic factors of UC. The 5-year survival rate of UC patients without muscle invasion is 80%, whereas the 5-year survival rate of patients presenting muscle invasion carcinoma is only 47% [1]. Approximately 70% of patients diagnosed with UC present non-invasive UC [2]. Nearly all non-invasive UCs are low-grade and present papillary proliferation pattern. The first-line therapy is the excision of tumors by TUR in combination with intravesical instillation of BCG. Unfortunately, many clinicians are confronted with early recurrence post initial treatment and a lack of clinically proven second-line therapies. To address this problem, numerous studies have been conducted; however, little experimental or clinical data exist to validate biomarkers associated with tumor occurrence/recurrence, progression, and metastasis. Therefore, it is of great importance to characterize the molecular mechanism of UC tumorigenesis for the development of new therapeutic strategies.

Nucleus accumbens-associated protein 1 (NACC1) is a member of the bric-a-brac tramtrack broad complex (also known as POZ; BTB/POZ) protein family, ubiquitously expressed in the brain, testis, and urinary bladder. NACC1 plays a regulatory role in several biological processes, including cellular proliferation, apoptosis, transcription regulation, and epigenetic reprogramming [3]. Expression of NACC1 is reported to be associated with poor prognosis of carcinomas in several organs, including the ovaries [4,5], oral cavity [6], colon/rectum [7,8,9], pancreas [10], uterine cervix [11], and prostate [12], as well as hematological [9] and melanocytic [13] neoplasms. In addition, NACC1 plays diverse roles in cancer biology, facilitating tumor migration and invasion through epithelial-mesenchymal transition (EMT) and inferring drug resistance. Several studies have shown that NACC1 has predictive value for cancer progression in ovary, uterus, and pancreatic ductal adenocarcinoma tumors [14,15].

In this study, we demonstrate that miR-331-3p in UC cells down-regulates NACC1 expression, which in turn inhibits cell proliferation by inducing senescence and cell cycle arrest in G0/G1 phase. In addition, suppression of NACC1 enhances tumor cell migration and invasion ability. Moreover, we found that NACC1 was up-regulated in non-invasive UC, but not in invasive tumors.

## 2. Results

### 2.1. NACC1 Is Overexpressed in Various Stages of UC in the Urinary Bladder 

**Figure 1:**
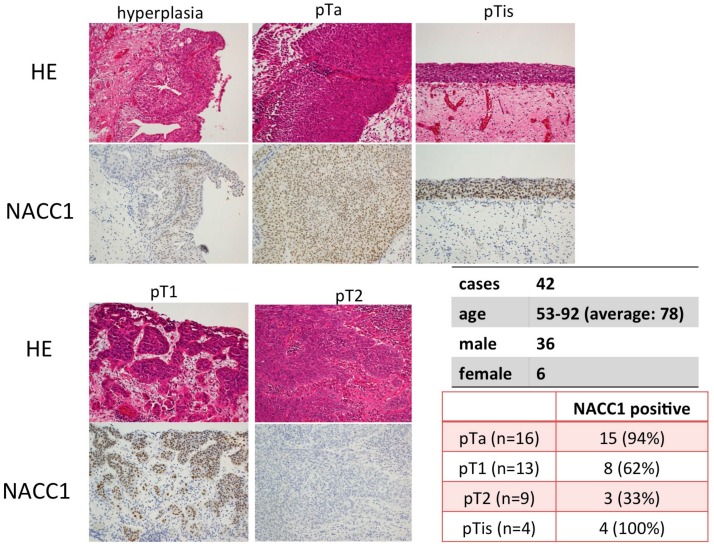
Expression of Nucleus accumbens-associated protein 1(NACC1) in Urothelial carcinoma (UC) of bladder tissues. UC were histologically classified in pTa, pTis, pT1 and >pT2. Hematoxylin and eosin (HE) stain and Immunohistochemistory,scale bar: ×200.

Using immunohistochemical analysis, we evaluated the expression of NACC1 in UC tissues of the urinary bladder. NACC1 was found to be localized in the nucleus of UC cells, and its expression was significantly higher in UC than in the normal or hyperplastic urothelium. Interestingly, NACC1 was expressed at significantly higher levels in low-grade, non-invasive UC and high-grade carcinoma in situ (pTis), compared with invasive UC (pT1 vs. pT2; *p* = 0.003). Contrasting results were obtained in invasive UC (pT1 or >pT2) (Figure 1) where expression levels were lower, but without any statistically-significant difference (*p* = 0.387). Other clinicopathological features, such as age (<=70, 71–80, 80<=) and gender, were not statistically associated with NACC1 expression. (age: *p* = 1.000. gender: *p* = 1.000).

### 2.2. NACC1 Inhibition Suppresses Proliferation in UC Cells

**Figure 2:**
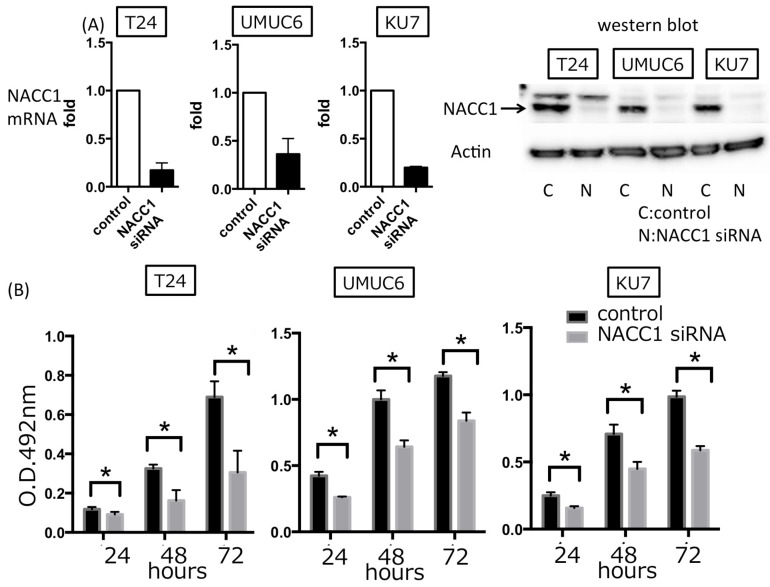
(**A**) The effect of transient NACC1 siRNA transfection in three UC cells. The left graph is qRT-PCR for NACC1 mRNA. The right image is western blot for NACC1 protein; (**B**) Methane thiosulfonate (MTS) assay in T24, UMUC6, and KU7 cells. Cell proliferation was suppressed by transient transfection of NACC1 siRNA (* *p* < 0.05).

**Figure 3:**
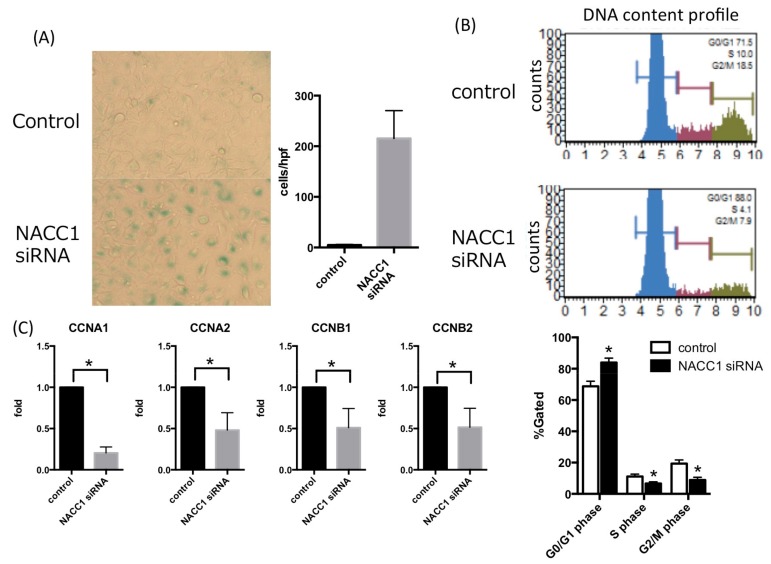
(**A**) Senescence-Associated β-Galactosidase (SA-β-gal) assay. Senescent cells with SA-β-galactosidase activity were significantly induced, scale bar: ×100; (**B**) The cell cycle assay for UC cells. Cell cycle arrest at G0/G1 phase was induced in T24 cells. (* *p* < 0.05); (**C**) mRNA expression of cyclin A and B under suppression of NACC1 in T24 cells. (* *p* < 0.05).

To evaluate the role of NACC1 in cell proliferation, we assessed the capacity of cell proliferation by transfecting the NACC1 siRNA into T24, UMUC6, and KU7 cells. We found that inhibition of NACC1 significantly suppressed cell proliferation, by approximately 60–70% after 72 h (Figure 2). To determine the mode of NACC1-induced regulation of proliferation in UC cells, cell senescence and cell cycle assays were performed and quantified by SA-β-gal and PI stains, respectively. As shown in Figure 3A,B, we found that silencing the NACC1 gene induced senescence and cell cycle arrest at the G0/G1 phase in T24 cells. In UMUC6 and KU7 cells, NACC1 gene silencing significantly induced cell senescence, whereas cell cycle arrest assays showed minor changes (average of G0G1 phase: UMUC6 control 65.64%, NACC1si RNA 67.72%, KU7 control 54.66%, NACC1 siRNA 50.60%) (Figure S1). Moreover, silencing NACC1 suppressed the expression of several cell cycle-related molecules, including cyclin A1 (CCNA1), cyclin A2 (CCNA2), cyclin B1 (CCNB1), and cyclin B2 (CCNB2) in T24 cells (Figure 3C). These findings suggest that NACC1 plays a key role in UC cell proliferation by regulating cell cycle and senescence. 

### 2.3. NACC1 Is One of the Putative Targets of MiR-331-3p

**Figure 4:**
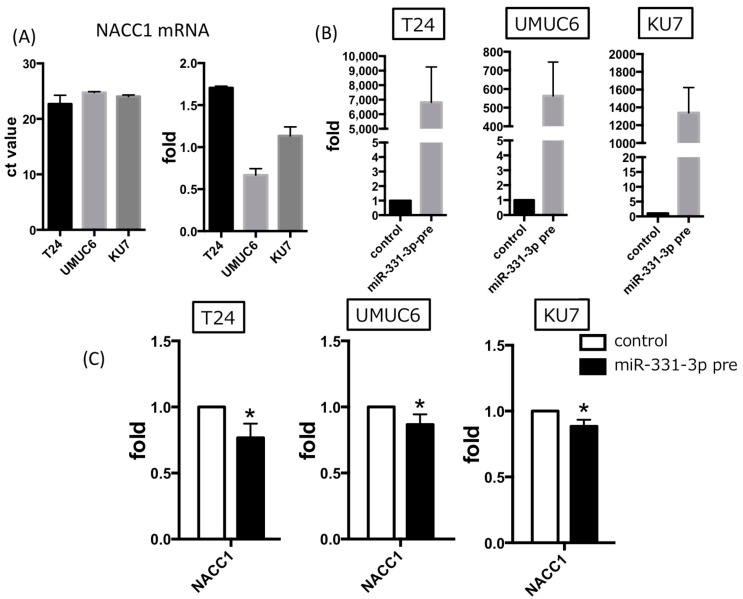
(**A**) mRNA expression of NACC1, which are the putative target molecules of miR-331-3p in T24, UMUC6, and KU7 cells. The left graph shows ct value of three UC cell lines. The right graph shows relative fold value (NACC1/actin); (**B**) The effect of miR-331-3p pre transfection by qPCR; (**C**) NACC1 expression under overexpression of miR-331-3p in three UC cell lines. (* *p* < 0.05, pre: precursor) miR-331-3p down-regulates NACC1 mRNA expression in T24, UMUC6, and KU7 cells.

**Figure 5:**
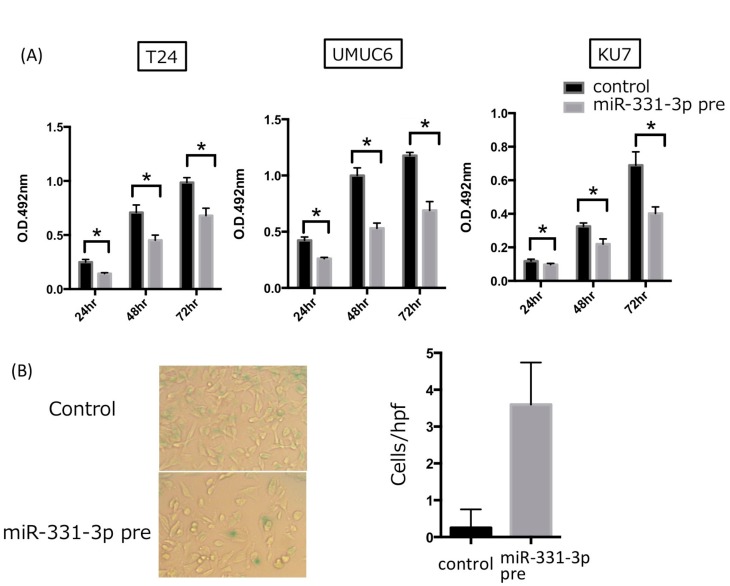
The effect of miR-331-3p on cell proliferation in three UC cell lines. (**A**) MTS assay. Cell proliferation was suppressed by overexpression of miR-331-3p. (* *p* < 0.05); (**B**) SA-β-gal assay for T24 cells. The Y-axis shows the number of positive cell counts per one high-power field (hpf). Positive cells were induced by transfection of miR-331-3p precursor (pre: precursor). Scale bar: ×100.

We have previously predicted several putative targets of miR-331-3p in silico using TargetScan analysis (release 6.2, June 2012) and miRSearch (V3.0, EXIQON). The predicted miR-331-3p and NACC1 interaction was detected using miRGate and DIANA TOOLS (Micro T-CDS) (Figure S2) [16]. In the present study, we determined whether miR-331-3p acts as the target of NACC1 in UC cells. Three UC cell lines showed a similar expression of NACC1 mRNA (Figure 4A). Overexpression of miR-331-3p resulted in significantly suppressed NACC1 mRNA and cell proliferation in the T24, UMUC6, and KU7 cell lines (Figure 4B and Figure 5A). In addition, we found that cell senescence was induced by miR-331-3p overexpression in T24 cells (Figure 5B). We assessed miR-331-3p expression levels in patients’ TUR-Bt samples. There was no significant difference in relative miR-331-3p/actin levels between non-invasive and invasive UC cells [average of non-invasive and invasive UC was 5.856 ± 2.131 and 2.921 ± 1.017, respectively (*p* = 0.2250)].

### 2.4. NACC1 Inhibits UC Cell Invasion and Migration In Vitro

**Figure 6:**
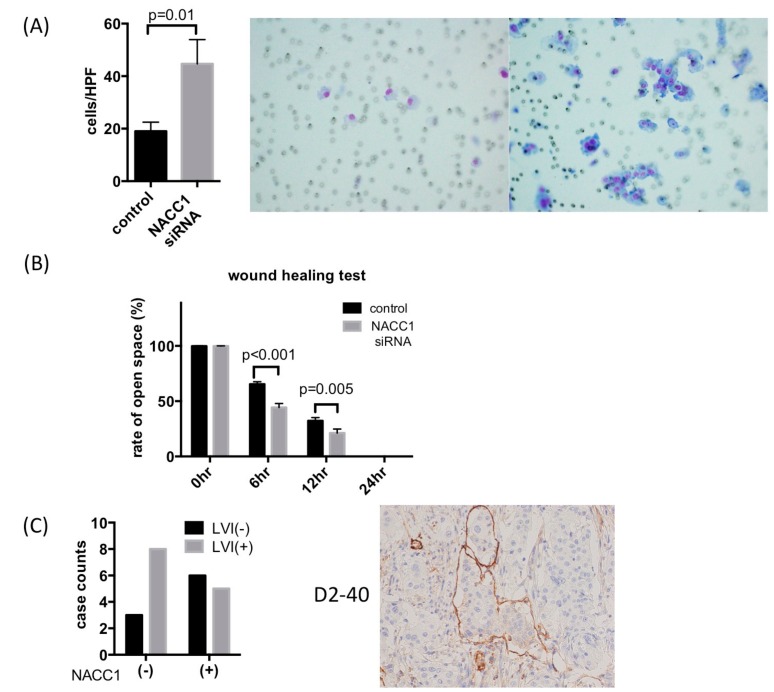
Cell invasion and migration assay in T24 cells. (**A**) Matrigel invasion assay showing promotion of invasion capability of T24 cells transfected with NACC1 siRNA; (**B**) Representative images and graphical display of rate of open space in wound closure (%) in T24 cells; (**C**) Immunohistochemistry for D2-40. In total, 22 cases of invasive UC were assessed. Scale bar: ×200.

As shown in Figure 1, NACC1 expression was lower in invasive UC than in non-invasive tumors (pTa and pTis). To evaluate the role of NACC1 with respect to invasive capability, we performed an invasion assay and wound healing test using T24 cells. Figure 6A indicates that Matrigel invasion capability of T24, UMUC6, and KU7 cells increased as a result of NACC1 inhibition (Figure 6A and Figure S1). In the wound healing test, control T24 cells were cultured to approximately 80% confluence and wounded. Subsequently, 6 and 12 h after the initial wound was made, the width of the wound had closed by approximately 65% and 44%, respectively. Motility was significantly increased in NACC1-suppressed cells (32% and 17% in 6 and 12 h, respectively) (Figure 6B and Figure S3). In invasive UC tissues, lymph and vessel invasion (LVI) significantly increased in NACC1-negative cases (Figure 6C). These results suggest that NACC1 suppression promotes the migration and invasion of UC cells.

## 3. Discussion 

We demonstrate for the first time that suppression of NACC1 contributes to the invasion capability while regulating cell proliferation by inducing cell cycle arrest at the G0/G1 phase and cell senescence in UC. 

One or more distinct signaling pathways of the cell cycle are controlled in the regulation of tumor progression. Some anticancer drugs contribute to inhibiting the checkpoint of the cell cycle. Cell cycle deregulation results in uncontrolled cell proliferation and severe alterations during the tumor development and progression. Therefore, the arrest of one or more phases of the cell cycle is regarded as an effective strategy for the chemotherapeutic treatment of tumors [17,18]. Conventional anticancer drug for UC therapy, such as vinblastine, doxorubicin, and cisplatin act during DNA synthesis and/or various phases of the cell cycle checkpoint, and produce anticancer effects [19]. There are several reports about anticancer drugs and biological compounds, such as desethylamiodarone [20], reversine [21], isoquercitrin [22], trichostatin A [23], and miconazole [24], for chemotherapy of UC. Moreover, the histone deacetylase (HDAC) inhibitor valproic acid (VPA), anticancer agent amygdalin and chalcone, which is a precursor compound for flavonoid synthesis in plants, induced cell growth inhibition via cell cycle arrest at G0/G1 or G2/M phase in UC [25,26,27]. The past gene expression study showed that multiple pathways associated with cell cycle progression and multiple core cell cycle components were up-regulated in grade 3 UC [28]. Several kinds of compounds which are related to regulation of the cell cycle may influence the function of NACC1. In the current study, NACC1 plays an important role in cell proliferation through the cell cycle and cell senescence. NACC1 may be one of key molecules in molecular targeting therapy. On the other hand, NACC1 suppress the invasion capability in invasive UC. From this point of view, NACC1 contributes to papillary growth and the inhibition of invasion to stroma in non-invasive UC. NACC1 may be a powerful molecular marker in the prognosis of UC. To confirm this hypothesis, we should assess prognostics, and/or survival analysis should be performed in a large cohort of UC patients in the future.

NACC1, the gene encoding NAC1, is one of the most significant genes to show a positive correlation between DNA and RNA copy number in human cancers [29], suggesting that it plays a driver role in cancer development. In fact, increased expression of NACC1 was found to be associated with disease aggressiveness, development of chemoresistance, and tumor recurrence in several types of human cancer, including ovarian, endometrial, and cervical carcinomas [14,30,31,32,33,34,35,36,37]. Experimentally, abundant NACC1 protein is essential for the migration and motility of cancer cells [35,38], maintenance of cell survival [32,34], prevention of cell senescence [39], and activation of autophagy in the presence of cisplatin through the high mobility group B1 pathway [40]. NACC1 is predominantly located in the nucleus and contains a nuclear localization signal [41]. A study of ovarian cancer indicated NACC1 as a driver gene, where it was amplified in approximately 20% of high-grade ovarian cancers, and was associated with p53 mutations [29,35]. Multiple evidence show that NACC1 is related to chemotherapeutic resistance and cancer recurrence, and affected overall survival and disease-free survival in ovarian cancer patients [15,29,32,33,39,40,42]. Studies indicate that NACC1 takes part in multiple processes to exert its action including growth, senescence, autophagy, epithelial-mesenchymal transition (EMT), to exert its action [33,35,43]. In the current study, we did not detect migration-related molecules. EMT-related molecules may contribute to UC progression which is coordinated with NACC1. We need to address the potential for biomarkers to improve treatment strategy. 

There are only a few specific markers for the diagnosis of UC, because the accurate molecular mechanism of UC carcinogenesis and progression have not been clearly determined. Thus, there has been a growing interest in clarifying the molecular function of candidate factors of UC carcinogenesis or cancer progression. Some miRNAs act as tumor suppressors, while others behave as oncogenes during cancer development and progression [44,45]. Our previous study indicated that miR-145 suppresses syndecan-1, up-regulates stem cell factors, induces cell senescence and differentiation, and increases squamous, glandular, and neuroendocrine markers in UC cells [46]. It is necessary to investigate the function of miRNAs and its target molecules by evaluating the expression of various stages and histological grades in UC to determine the candidate molecular marker for diagnosis. In this study, we demonstrated that NACC1 was important to an invasive mechanism of the UC as a candidate target of miRNA-331-3p, and determined that miRNA-331-3p and NACC1 regulated cell proliferation via cell cycle arrest at G0/G1 phase and cell senescence. 

NACC1 protein expression decreased in invasive UC, and the in vitro experiment showed that invasion and migration ability were enhanced by the suppression of NACC1. These results suggest that NACC1 has an important role in cancer progression, i.e., by negatively regulating cell migration and invasion. We have previously reported that syndecan-1 (CD138) up-regulates miR-331-3p expression by directly targeting neuropilin 2 (NRP2) and NACC1 to mediate the EMT in prostate cancer cells [12]. Consistent with our previous report, we show that NACC1 is associated with cell migration and invasion. 

**Figure 7:**
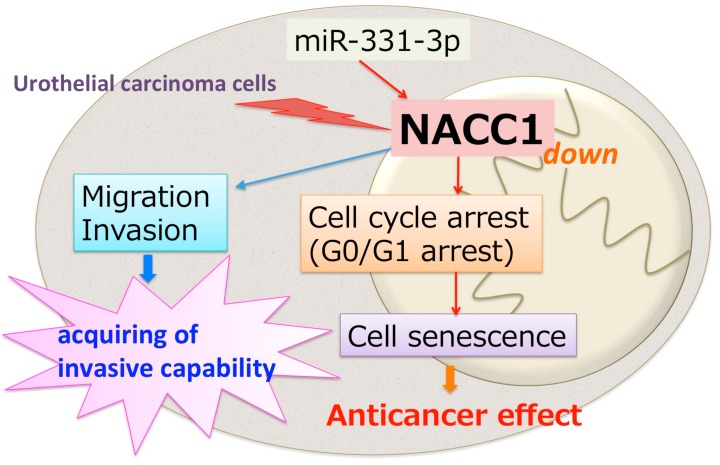
Suppression of NACC1 induces cell cycle arrest at G0/G1 phase and cell senescence in UC. On the other hand, NACC1 promotes cell migration and invasion capability.

In conclusion, these findings show that overexpression of miR-331-3p, and subsequently, inhibition of NACC1, resulted in the suppression of UC cell proliferation, but could lead to cell migration and invasion. NACC1 plays an important role in the progression of UC (Figure 7). In addition, NACC1 and miR-331-3p may be useful clinical diagnostic and/or prognostic markers of UC.

## 4. Materials and Methods

### 4.1. Cell Lines

Research in this study was conducted on human cancer cell lines, following procedures in accordance with the ethical standards formulated in the Declaration of Helsinki. The human UC cell lines, T24 and UMUC6 were purchased from American Type Culture Collection (Manassas, VA, USA). KU7 was derived from human papillary bladder cancer [47]. T24, UMUC6, and KU7 were cultured in RPMI 1640 media supplemented with 10% fetal bovine serum and 50 units/mL penicillin-streptomycin at 37 °C in 5% CO_2_.

### 4.2. siRNA and miRNA Inhibitor/Precursor Transfection in UC Cells

T24, UMUC6, or KU7 were seeded at a density of 1 × 10^4^ cells/well and transfected for 72 h with 100 ng/L siRNA against NACC1 or 100 pg of Pre-miR^TM^ miRNA Precursor (hsa-miR-331-3p Life Technologies, Carlsbad, CA, USA). Transfection was performed in Lipofectamine RNAiMAX (Life Technologies, Carlsbad, CA, USA), according to the manufacturer’s protocol. The sequence of the NACC1 siRNA was 5′-CCGGGTCCACTTCCATTGTTA-3′. We used AllStars Negative Control siRNA (QIAGEN, Venlo, Netherlands.) as control for a transient siRNA or miRNA precursor in both transfection experiments.

### 4.3. Quantitative RT-PCR Analysis of miRNA and mRNA

For purification of total RNA from cells, including miRNA, we used the miRNeasy Mini kit (QIAGEN, Venlo, The Netherlands). For quantitative RT-PCR, first-strand cDNA was synthesized from 1 g of total RNA using the PrimeScript RT Master Mix (Perfect Real Time) and SYBR Premix Ex Taq II (TliRNaseH Plus) (TaKaRa, Otsu, Japan). Quantitative PCR (qPCR) thermal cycling parameters were 95 °C for 30 s, followed by 55–63 °C for 30 s, for a total of 35–45 cycles. The PCR primers were as follows:

NACC1 sense 5′-CTCTCCCGGCTGAACTTATCAAC-3′,
NACC1 antisense 5′-GTACACGTTGGTGCCTGTCAC-3′;
Actin sense 5′-CTCTTCCAGCCTTCCTTCCT-3′,
Actin antisense 5′-AGCACTGTGTTGGCGTACAG-3′;
CCNA1 sense 5′-ACCCCAAGAGTGGAGTTGTG-3′,
CCNA1 antisense 5′-GGAAGGCATTTTCTGATCCA-3′;
CCNA2 sense 5′-TTATTGCTGGAGCTGCCTTT-3′,
CCNA2 antisense 5′-ACTGTTGTGCATGCTGTGGT-3′;
CCNB1 sense 5′-CGGGAAGTCACTGGAAACAT-3′,
CCNB1 antisense 5′-AAACATGGCAGTGACACCAA-3′;
CCNB2 sense 5′-TGGAAAAGTTGGCTCCAAAG-3′,
CCNB2 antisense 5′-CCTCCAGCTGCCTGAGATAC-3′.

### 4.4. Cell Proliferation Assay

For the cell proliferation assay, a methane thiosulfonate (MTS) reagent was used, as previously described [48]. All experiments were performed in triplicate.

### 4.5. Senescence-Associated β-Galactosidase (SA-β-gal) Assay

SA-β-gal activity was measured using the Senescence Detection Kit (BioVision, San Francisco, CA, USA) in T24, UMUC6 and KU7 cells transfected for 72 h with negative control siRNA, miR-331-3p precursor or NACC1 siRNA. The assay was performed in accordance with the manufacturer’s instructions. 

### 4.6. Cell Invasion Assay and Wound Healing Assay

In vitro invasion assays were performed using Corning Biocoat Matrigel Invasion Chambers (Corning, Bedford, MA, USA) according to manufacturer’s instructions. T24 cells were seeded at 2.5 × 10^4^ cells per well in a 24-well dish, and transfected with 100 nmol/L NACC1 siRNA or negative control siRNA for 24 h using Lipofectamine RNAiMAX (Life Technologies, Carlsbad, CA, USA), in accordance with the manufacturer’s protocol. After culturing for the indicated time, the samples were removed and re-plated in the Matrigel chambers. After culturing for 48 h, invading cells were stained and counted under a light microscope. The experiment was repeated three times. For the cell migration assay, T24 cells were plated in six-well dishes at 1 × 10^5^ cells per well and transfected with 100 nmol/L NACC1 siRNA or negative control siRNA for 24 h. Cells that had received negative control siRNA treatment and the NACC1 siRNA-transfected cells were scratched using a 200 mL pipette tip. The wound image was captured at time 0. The cells were incubated, and the wound areas were visualized at 6, 12, and 24 h. The extent of wound closure was measured.

### 4.7. Tissue Samples 

We examined 42 transurethral resections of UC specimens that did not undergo chemotherapy or Bacillus Calmette-Guerin treatment (aged 53–92 years). The present study received ethics committee approval from Nara Medical University (NMU900). Informed consent was obtained from all patients participating in this study. All tissue samples were fixed in 10% formalin for 48 h and processed through graded alcohols to paraffin. Paraffin blocks were sectioned at 3 µm intervals and stained with hematoxylin and eosin (HE) for histological diagnosis. Tumor stage and grade were noted at the time of diagnosis by two independent urological pathologists (TF and CO). 

### 4.8. Immunohistochemistry

Sections were incubated with the primary antibody against NACC1, D2-40 and CD31 for one hour at room temperature, and the reactions were visualized using a Histofine kit (Nichirei, Tokyo, Japan) using diaminobenzidine as the chromogen, with hematoxylin counterstaining. We defined a positive NACC1 staining as our criteria for the cut off of NACC1 immunostaining.

### 4.9. Statistical Analysis

Statistical analysis of the experimental data was performed with GraphPad Prism 6.0 (GraphPad Software, La Jolla, CA, USA). The software program used the t-test to compare the two groups. All data are expressed as the mean ±standard error of the mean (SEM). A *p* value of <0.05 was considered significant. The statistical analysis of clinical samples was performed using the Chi-square test (Fisher’s exact test).

## 5. Conclusions

In conclusion, the inhibition of NACC1 resulted in suppression of UC cell proliferation by inducing cell cycle arrest at G0/G1 phase and cell senescence, but could lead to cell migration and invasion. The findings above suggest that NACC1 plays an important role in the progression of UC. Furthermore, the results reported here indicate that NACC1 can serve as a potential biomarker for the prediction and prognosis of UC, and thus, contribute to effective treatment strategies.

## Supplementary Materials

The following are available online at http://www.mdpi.com/2072-6694/10/10/347/s1, Figure S1: Images of SA-β-gal assay and cell invasion assay in KU7 and UMUC6, Figure S2: In silico analysis of NACC1 and miR-331-3p interaction, Figure S3: Images of wound healing test in T24 cells.
