# Supplementary material for: NACC1, as a Target of MicroRNA-331-3p, Regulates Cell Proliferation in Urothelial Carcinoma Cells
Source: Cancers (Basel). 2018 Sep 21;10(10):347. doi: 10.3390/cancers10100347 (PMC6210667; doi:10.3390/cancers10100347)
Supplement: Supplementary file 1 [file cancers-10-00347-s001.pdf]

Supplementary Materials: NACC1, As A Target of MicroRNA-331-3p, Regulates Cell Proliferation in Urothelial Carcinoma Cells

Kohei Morita, Tomomi Fujii, Hiroe Itami, Tomoko Uchiyama, Tokiko Nakai, Kinta Hatakeyama, Aya Sugimoto, Makito Miyake, Yasushi Nakai, Nobumichi Tanaka, Keiji Shimada, Masaharu Yamazaki, Kiyohide Fujimoto and Chiho Ohbayashi

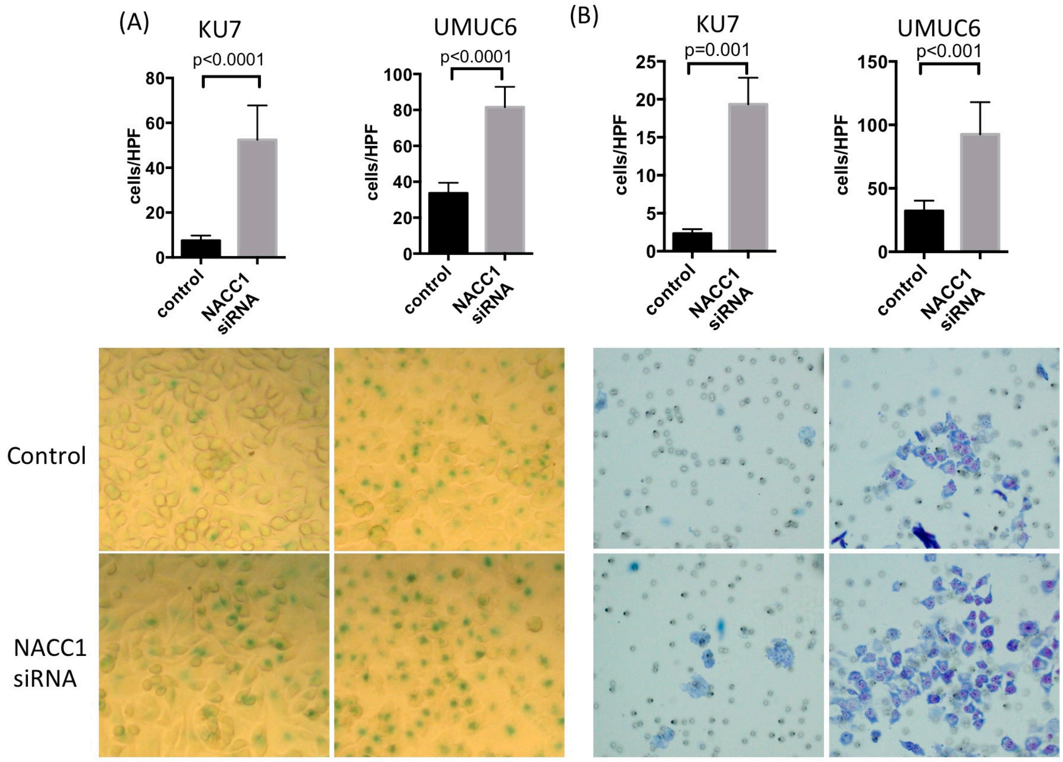

Figure S1. Images of SA-β-gal assay and cell invasion assay in KU7 and UMUC6. (**A**) SA-β-gal assay for UMUC6 and KU7 cells transfected with NACC1 siRNA. The Y-axis shows the number of positive cell counts per one hpf; (**B**) Matrigel invasion assay showing promotion of invasion capability of UMUC6 and KU7 cells transfected with NACC1 siRNA. Scale bar: ×100.

DIANA TOOLS – MicroT-CDS
(Transcript)Homo sapiens nucleus accumbens associated 1 (NACC1), mRNA (NM_052876).
(miRNA) miR-331-3p

```
                  2035
(Transcript) 5' UCUCCACACUCACC_____CCA_________A 3'
                ______________AGGC____CCAGGGG_
                ______________UCCG____GGUCCCC _
(miRNA) 3'      _____________A_________________G 5'

                  2153
(Transcript) 5' CCUUGGGA__UG__________C_________U 3'
                ________CU__AGGG_GGG_CCCAGGGG_
                ________GA__UCCU_UCC _GGGUCCCC_
(miRNA) 3'      _______A_________A______________G 5'

                  2570
(Transcript) 5' CAGUAGGAC______UU___AA_________A 3'
                _________CUGGG___GG__CCCAGGGG_
                _________GAUCC___CC __GGGUCCCC_
(miRNA) 3'      ________A_____UAU_____________G 5'

                  2765
(Transcript) 5' GUCUAUUGUCCCC__C __ UC___________ U 3'
                _____________ CU_GG_____ CCCAGGGG_
                              GA_CC_____ GGGUCCCC_
(miRNA) 3'      ____________ A__U__UAUCC ________ G 5'

                  3292
(Transcript) 5' ACCCCCACCUACCCACUGCU_________ U 3'
                ___________________CCCAGGGG_
                ___________________GGGUCCCC_
(miRNA) 3'      ___________________C_________G 5'

                  3304
(Transcript) 5' CCACUG__CCC_____GUCUC___________ 3'
                ______CU___AGGG______UCCAGGGGC
                ______GA___UCCU______GGGUCCCCG
(miRNA) 3'      _____A__________AUCC____________ 5'

                  3683
(Transcript) 5' GCGG_____CCUGACGG_____G_______ 3'
                ____CUGGG________AGGCC_AGGGGC
                ____GAUCC________UCCGG_UCCCCG
(miRNA) 3'      ___A_____UA____________G_______ 5'

                  3982
(Transcript) 5' GGGGGUG____GAA_____GGG_______G 3'
                _______GGGA___GGGUC___CAGGGG_
                _______UCCU___UCCGG___ GUCCCC_
(miRNA) 3'      ______A____A__________________G 5'

                  3991
(Transcript) 5' GAGA______CGGGCAGG_____G_______A 3'
                ____AGGGU__________GGGU_CAGGGG_
                ____UCCUA__________ UCCG_ GUCCCC_
(miRNA) 3'      ___A______________________G_______G 5'
```

Figure S2. *In silico* analysis of NACC1 and miR-331-3p interaction.

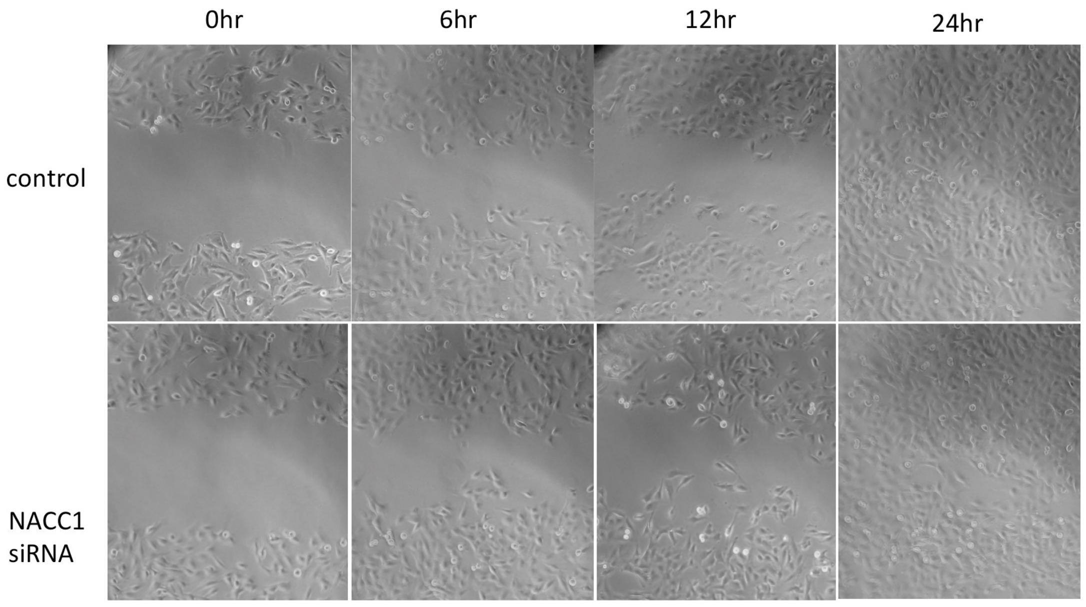

Figure S3. Images of wound healing test in T24 cells. Scale bar: ×100.
